# Continuous Butanol Fermentation of Dilute Acid-Pretreated De-oiled Rice Bran by *Clostridium acetobutylicum* YM1

**DOI:** 10.1038/s41598-019-40840-y

**Published:** 2019-03-15

**Authors:** Najeeb Kaid Nasser Al-Shorgani, Abdualati Ibrahim Al-Tabib, Abudukeremu Kadier, Mohd Fauzi Zanil, Kiat Moon Lee, Mohd Sahaid Kalil

**Affiliations:** 1 Department of Chemical and Process Engineering, Faculty of Engineering and Built Environment, Universiti Kebangsaan Malaysia, 43600 UKM Bangi, Selangor Malaysia; 2 Department of Applied Microbiology, Faculty of Applied Sciences, Taiz University, 6803 Taiz, Yemen; 3 Department of Chemical and Petroleum Engineering, Faculty of Engineering & Built Environment, UCSI University, 56000 Kuala Lumpur, Malaysia

## Abstract

Continuous fermentation of dilute acid-pretreated de-oiled rice bran (DRB) to butanol by the *Clostridium acetobutylicum* YM1 strain was investigated. Pretreatment of DRB with dilute sulfuric acid (1%) resulted in the production of 42.12 g/L total sugars, including 25.57 g/L glucose, 15.1 g/L xylose and 1.46 g/L cellobiose. Pretreated-DRB (SADRB) was used as a fermentation medium at various dilution rates, and a dilution rate of 0.02 h^−1^ was optimal for solvent production, in which 11.18 g/L of total solvent was produced (acetone 4.37 g/L, butanol 5.89 g/L and ethanol 0.92 g/L). Detoxification of SADRB with activated charcoal resulted in the high removal of fermentation inhibitory compounds. Fermentation of detoxified-SADRB in continuous fermentation with a dilution rate of 0.02 h^−1^ achieved higher concentrations of solvent (12.42 g/L) and butanol (6.87 g/L), respectively, with a solvent productivity of 0.248 g/L.h. This study showed that the solvent concentration and productivity in continuous fermentation from SADRB was higher than that obtained from batch culture fermentation. This study also provides an economic assessment for butanol production in continuous fermentation process from DRB to validate the commercial viability of this process.

## Introduction

The worldwide energy demand is continuously increasing over time due to the expected decline of petrol and due to environmental issues that are related to the use of petrol as a source of energy^1^. Petroleum oil is a non-renewable resource and is going to be depleted soon. Accordingly, it is necessary to find renewable alternative sources of fuel that can substitute for oil and are environmentally friendly. One of the best liquid biofuels that can substitute for gasoline is butanol, which has similar properties as gasoline.

Butanol is produced biologically by acetone-butanol-ethanol (ABE) fermentation using solventogenic *Clostridium* species*. Clostridium acetobutylicum* YM1 is a solvent-producing strain that was isolated from local agricultural soil in Malaysia and has been used for butanol and hydrogen production^2,3^.

The substrate cost, microbial strain performance, fermentation process mode and recovery process significantly affect the economics of butanol production. The use of low cost and sustainable feedstocks for butanol production can minimize the cost of this process^4^. As reported in the literature, the most influential factor in ABE fermentation is the cost of the substrate, which constitutes approximately 60% of the total process cost^5^. Hence, exploring less expensive substrates for ABE fermentation is essential for making ABE fermentation economically viable. Agricultural biomass residues are a suitable alternative because of its low price feedstocks. However, prior to utilizing lignocellulosic feedstocks, they require pretreatment and saccharification.

Developing excellent strains that are resistant to butanol toxicity and hyper-butanol producing is an ideal idea for improving butanol fermentation, but it still needs more efforts. Some *Clostridium* strains have been engineered using systematic or mutagenesis approaches to improve butanol productivity and overcome the butanol toxicity. Earlier, mutagenesis and genetic manipulation methods such as homologous recombination and antisense RNA were used to understand gene functions and enhance butanol production. A new technique called CRISPR-Cas provided large-scale genome editing of *Clostridium* over than mutagenesis and genetic manipulation techniques. CRISPR-Cas-based editing tool kits is also a promising biotechnology which can be used for efficient *Clostridium* cell engineering for improving butanol production^6^. A comprehensive review on recent strategies for strain development and advanced downstream process techniques for butanol production by *Clostridium acetobutylicum* is detailed by Xue *et al*.^6^.

Batch culture fermentation of butanol production by ABE fermentation is the most practiced fermentation for butanol production, while in industrial large scale of butanol production, the continuous fermentation mode is more productive than the batch fermentation mode. There are many shortcomings in the batch fermentation operation of butanol including the accumulation of butanol which halts the fermentation due to the toxicity, the period required for medium preparation and bioreactor sterilization during which processes are ceased, considerable down time and low yield and productivity.

Butanol production by continuous fermentation prevents the accumulation of butanol and then eliminates the cytotoxicity of butanol. The feeding of fresh medium and the elimination of product accumulation is a useful method that can keep the operation going at a steady rate which results into significant enhancement in the butanol yield and productivity. Continuous ABE fermentation has several benefits compared to batch fermentation, including higher productivity, less product inhibition, and less downtime, while there are some difficulties associated with continuous ABE fermentation, such as the two-phase nature of ABE fermentation (acidogenesis and solventogenesis), phage contamination and flocculation of bacterial growth, which can make steady state fermentation unstable. The solvent productivity in the batch ABE fermentation process is usually low while the solvent productivity in continuous fermentation is greater, which makes continuous ABE fermentation more attractive for commercial industrial ABE production. In continuous ABE fermentation, glucose or corn starch are mainly consumed as the major feedstock^7^.

Cell immobilization of clostridia is an efficient approach to obtain a high productive continuous fermentation system for butanol production. Cell immobilization protects the cells from butanol toxicity and prevents them from bleeding during continuous fermentation. Butanol production using an immobilized cell of *Clostridium sp*. in continuous fermentation systems improved the fermentation productivity and stability^8^. Due to the biphasic of ABE fermentation, it was found that a single chemostat bioreactor is not applicable to operate continuous fermentation for high productivity of butanol and tanks-in-series systems were suggested as an option for high efficient system using sustainable feedstocks and efficient microbial strains^9^.

Integrated butanol recovery techniques such as gas stripping, pervaporation, liquid-liquid extraction and adsorption could remove butanol simultaneously during the ABE fermentation, reduce the butanol toxicity and subsequently increase fermentation productivity^10^. Advanced integrated recovery techniques for *in situ* butanol separation with using an engineered microbial strain could also improve the efficiency and stability of butanol production, which was proposed to make this process viable economically^6^. The conventional recovery technique for butanol is distillation which is characterized to be high-energy consumption and not economically competitive whereas *in situ* butanol recovery technologies are energy-saving and can be applied during the fermentation to reduce the product toxicity and improve butanol productivity^10,11^.

Rice is the staple food of more than 3.5 billion people and the worldwide production of rice is expected to reach 480.1 million metric tons in 2017^12^. Rice bran is a residual waste of the rice processing industry that accounts for approximately 10% of rice production. Rice bran is rich in oil and the waste of oil from extraction is called de-oiled rice bran (DRB). DRB is available, is inexpensive, contains large amounts of carbohydrates and has limited application as an animal feed. Therefore, DRB is a potential substrate for an economically viable butanol production process^13^.

Prior to the bioconversion of agricultural residues to butanol by *Clostridium*, a pretreatment/hydrolysis step is required to release fermentable sugars, which can then be utilized by *Clostridium* strains for butanol production^14^. Various pretreatment approaches, including physical and chemical methods or a combination of the two methods, have been applied on agricultural biomass to produce fermentable sugars^15^. The most common pretreatment method used for the pretreatment of agricultural biomass is dilute sulfuric acid, in which the agricultural biomass is exposed to high temperature and dilute sulfuric acid.

During the pretreatment process of lignocellulosic biomass, a number of inhibitor compounds are usually produced as a result of extreme degradation. These fermentation inhibitory compounds are including furfural, hydroxymethylfurfural (HMF), acetic, formic, ρ- coumaric, ferulic, levulinic, glucuronic acids, and phenolic compounds that inhibit bacterial growth and then negatively affect the butanol fermentation efficiency^16^. Many methods have been applied to remove or decrease these inhibitory compounds, including the use of adsorbent resin and activated charcoal, the dilution of hydrolysate, overliming and the development of tolerant microbial strains^13,17,18^.

In the current study, continuous fermentation for butanol production was explored using dilute sulfuric acid pretreated-DRB as a fermentation medium by *C. acetobutylicum* YM1. The butanol production from SADRB by the strain YM1 in a continuous fermentation process was carried out at different dilution rates and using non-detoxified and detoxified SADRB hydrolysate.

## Results and Discussion

### Batch fermentation of SADRB

**Figure 1:**
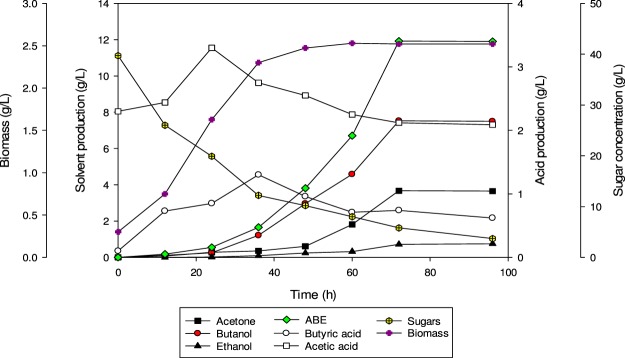
Butanol production in batch culture fermentation of SADRB by *C. acetobutylicum* YM1.

Batch fermentation experiments with SADRB using *C. acetobutylicum* YM1 were conducted as a control to compare the fermentation performance. The initial concentration of total sugars was 39.7 g/L and the fermentation was started by inoculating the medium with 10% (v/v) fresh inoculum of *C. acetobutylicum* YM1. In this experiment, the maximum production of butanol and ABE was obtained after 72 h, at 7.53 g/L and 11.92 g/L, respectively. Acetone and ethanol concentrations also reached their maximum at 72 h (Fig. 1). The sugar was mostly consumed, and only 8 g/L was left after 72 h of fermentation time. The ratio of butanol to acetone was 2: 1, which is a typical ratio in ABE fermentation as reported in the literature^19^. The yields of butanol and ABE were 0.25 g/g and 0.38 g/g, respectively, and the production rates of butanol and ABE were 0.105 g/L.h and 0.166 g/L.h, respectively. The butyric acid concentration reached a maximum value (1.3 g/L) at 36 h, while acetic acid was maximized at 24 h. Then, both acids were utilized during the solventogenic phase, with final concentrations of butyric and acetic acids of 0.74 g/L and 2.12 g/L, respectively, after 72 h of fermentation. Butyric acid utilization was clearly associated with butanol triggering, which is initiated once the bacterial growth reaches a stationary phase as a secondary metabolite (Fig. 1). In comparison, 9.66 g/L of total ABE with 6.75 g/L of butanol was produced from fermentation with SADRB (33.4 g/L sugar) by *C. saccharoperbutylacetonicum* N1-4, with an ABE yield and productivity of 0.35 g/g and 0.081 g/L.h, respectively^13^.

### Batch fermentation of detoxified SADRB

SADRB hydrolysate was detoxified by activated charcoal to reduce or remove inhibitory compounds from the hydrolysate, including furfural, HMF, acetic acid, formic acid and levulinic acid. The concentrations of fermentation inhibitors before detoxification and after detoxification by charcoal are listed in Table 1.

**Table 1:** Characteristic of non-detoxified SADRB and detoxified SADRB by activated charcoal.

Fermentation Inhibitors	Non-detoxified SADRB	Detoxified SADRB	Reduction (%)
Total Sugars (g/L)	42.12	40.17	4.63
Furfural (g/L)	0.33	0.0012	99.64
HMF (g/L)	0.46	0.0014	99.69
Acetic acid (g/L)	2.70	1.30	51. 85
Formic acid (g/L)	0.69	0.10	85.51
Levulinic acid (g/L)	1.21	0.59	50.85

Activated charcoal showed a high potential for reducing fermentation inhibitors from SADRB hydrolysate, and a nonsignificant concentration of sugars was reduced (4.63%), as shown in Table 1. The removal efficiencies of furfural, 5-HMF, acetic acid, formic acid and levulinic acid were 99.64, 99.69, 51.85, 85.51 and 50.85%, respectively.

Activated charcoal can be regenerated after being used in detoxification to reduce the cost of the process. The most common method used for the regeneration of activated charcoal is thermal regeneration. Moreover, gasification by air, CO_2_ or nitrogen, heating by microwave, pyrolysis and wet oxidation techniques have also been applied for the regeneration of activated charcoal^20–22^. Regeneration of activated charcoal had some benefits such as reducing the use of the coal and the natural resources, reducing the pollution caused by the waste of the used activated charcoal. In addition, the energy required to regenerate activated charcoal is less than what is needed to produce new activated charcoal^22^. However, some studies have reported poor regeneration efficiency of activated charcoal due to the oligomerization of phenolic compounds. The irreversible adsorption of phenolic compounds onto activated charcoal represents a major problem which reduces the lifetime of the activated charcoal usage, increases the operation cost and contributes to the pollution due to the disposal of the used activated charcoal. Though, phenol-loaded activated charcoal was regenerated by electrochemical regeneration technique with 80% of regeneration efficiencies^23^. Moreover, a special activated charcoal was developed for hampering oligomerization of phenolic compounds on its surface by controlling the activation process for obtaining high microporosity^24^.

It was noticeable that the concentration of acetic acid in the SADRB hydrolysate was high (2.5 ± 0.2 g/L). Acetic acid is released from the hydrolysis of hemicellulosic materials that contain many acetyl groups. It was reported that acetic acid has an inhibitory impact on the biomass concentration of *C. acetobutylicum* at high concentrations (0.19 M)^25,26^. Therefore, as a detoxification response, *C. acetobutylicum* converts acetic acid to acetone in the solventogenic phase through an enzymatic system^27^.

Detoxification of SADRB hydrolysate by activated charcoal resulted in a significant decrease in the acetic acid portion (51.85%). Moreover, dilute sulfuric acid pretreatment of DRB released 0.69 g/L of formic acid. Previously, it was found that the presence of formic acid in the fermentation medium led to a high reduction of butanol production by *C. acetobutylicum*, that ABE production was reduced to 77% in the presence of 1 g/L formic acid and that there was a 25% reduction upon addition of 0.4 g/L formic acid^26^. Furthermore, Wang *et al*. reported that the addition of 0.046 g/L of formic acid to corn mash medium with *C. acetobutylicum* caused an acid crash during ABE fermentation and this effect of formic acid on ABE fermentation might be mediated through oxidative stress^28^. In a toxicity test, we found that the addition of 1 g/L formic acid to the medium of *C. acetobutylicum* YM1 resulted in total growth inhibition (data will be published elsewhere). Interestingly, detoxification of SADRB hydrolysate by activated charcoal decreased the concentration of formic acid to 0.1 g/L (85.51%), which showed the considerable removal efficiency of charcoal.

In the literature, it was found that detoxification of hardwood Kraft black liquor hydrolysate using activated charcoal could recover 99–100% of xylose^29^, which means that negligible xylose was lost during detoxification, which is in agreement with our results. Moreover, Mussatto and Roberto^30^ and Kamal *et al*.^31^ reported that activated charcoal has a high ability to adsorb fermentation inhibitor compounds with less reduction of the sugar concentration^30,31^. In a study conducted by Guo *et al*., it was found that detoxification of spruce hydrolysate by activated charcoal resulted in the removal of 94% furfural and HMF^32^.

**Figure 2:**
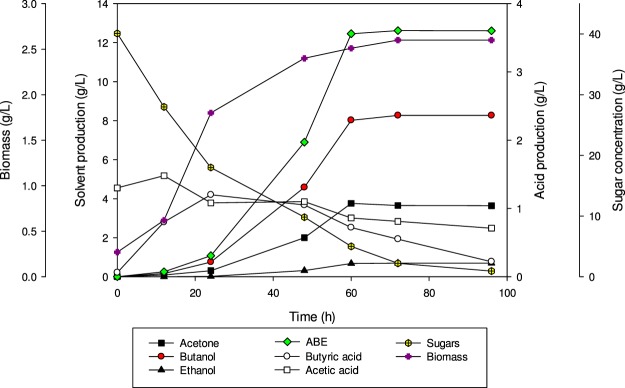
Butanol production in batch culture fermentation of detoxified SADRB by *C. acetobutylicum* YM1.

Batch fermentation of detoxified SADRB was used for butanol production by *C. acetobutylicum* YM1. The starting sugar concentration was 40.1 g/L and only 0.92 g/L of residual sugar remained in the culture after 96 h of fermentation. The maximum total ABE, butanol, acetone and ethanol obtained after 72 h of batch fermentation was 12.62, 8.27, 3.65 and 0.7 g/L, respectively (Fig. 2). The ABE and butanol produced from detoxified SADRB were higher than that produced when non-detoxified SADRB was used in batch fermentation with *C. acetobutylicum* YM1 under similar conditions. The productivities of butanol (0.115 g/L.h) and total ABE (0.175 g/L.h) in this experiment were also higher than when non-detoxified of SADRB was employed. The results showed that detoxified SADRB produced higher concentrations of solvents compared to that produced from non-detoxified SADRB.

### Continuous fermentation of glucose

**Figure 3:**
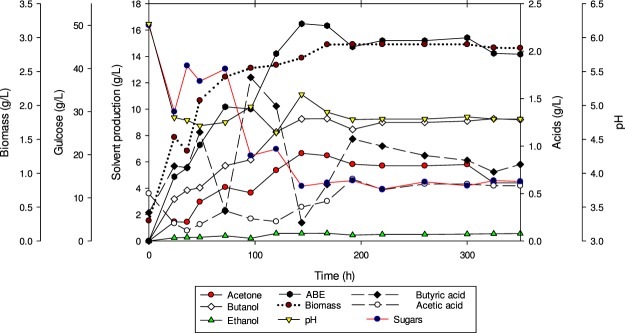
Profiles of continuous fermentation for butanol production from glucose (5%) using *C. acetobutylicum* YM1 with a dilution rate of 0.05 h^−1^.

Continuous fermentation using 50 g/L of glucose at a dilution rate of 0.05 h^−1^ was conducted as a control test. Figure 3 represents the continuous fermentation profile of butanol production by *C. acetobutylicum* YM1. The steady state was reached after 120 h of fermentation and continued afterward. The glucose consumption was maintained between 12–14 g/L under steady-state fermentation when the dilution rate was 0.05 h^−1^.

The average total ABE production at the steady state was 16.51 g/L with butanol, acetone and ethanol concentrations of 9.28, 6.65 and 0.58 g/L, respectively. The biomass concentration was also kept constant in the stationary phase, and no decline of biomass concentration was observed during 350 h of continuous fermentation (Fig. 3). The butyric acid concentration increased up to 1.14 g/L after 48 h and after that, the concentration was decreased and kept constant, with an average concentration of 0.8 ± 0.2 g/L, while the concentration of acetic acid was also constant at steady state with a concentration of 0.5 ± 0.1 g/L. Culture pH was not controlled in this study and was constant after 24 h of fermentation at pH 4.8 ± 0.1 during the whole fermentation time. In this experiment, the productivities of total solvent and butanol were 0.823 and 0.464 g/L.h, respectively. The yields of ABE and butanol in the continuous fermentation of butanol from glucose by *C. acetobutylicum* YM1 were 0.48 g/g and 0.25 g/g, respectively.

In comparison, the ABE productivity in continuous fermentation with 5% (w/v) glucose by *C. acetobutylicum* YM1 was higher than that found when batch fermentation was performed using *C. acetobutylicum* YM1 in 5% (w/v) glucose, representing an 87.1% increase. Batch fermentation of 5% glucose resulted in 12.72 g/L total solvents in 120 h, including 3.09 g/L acetone, 9.48 g/L butanol and 0.16 g/L ethanol. Therefore, the results showed that continuous fermentation of 5% glucose by *C. acetobutylicum* YM1 was superior in solvent production and productivity compared to batch culture fermentation.

Liew *et al*., reported lower solvent production (9.1 g/L) compared to our study when they operated a continuous fermentation by *Clostridium saccharobutylicum* DSM 13864 at a dilution rate of 0.05 h^−1^ ^33^. In addition, the solvent productivity (0.823 g/L.h) obtained in this study was higher compared to that reported by Liew *et al*., at 0.46 g/L.h^33^.

### Continuous fermentation of SADRB

**Figure 4:**
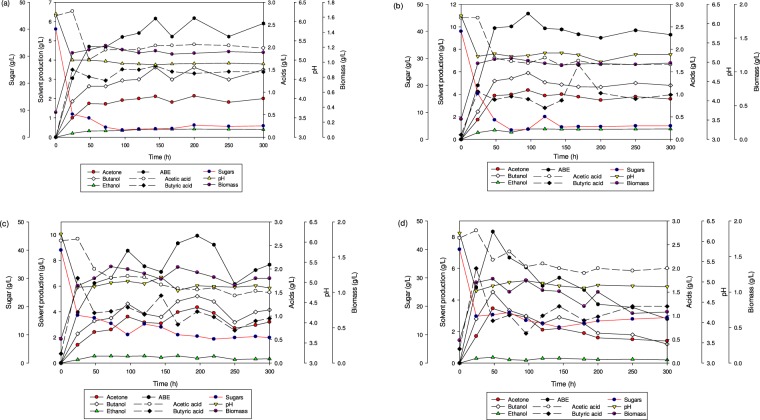
Continuous fermentation profiles of butanol production from SADRB using *C. acetobutylicum* YM1; (**a**) at a dilution rate of 0.01 h^−1^, (**b**) at a dilution rate of 0.02 h^−1^, (**c**) at a dilution rate of 0.03 h^−1^ and (**d**) at a dilution rate of 0.05 h^−1^.

SADRB was used as a fermentation medium for the continuous fermentation of butanol by *C. acetobutylicum* YM1. Various dilution rates were applied: 0.01, 0.02, 0.03 and 0.05 h^−1^. Profiles of continuous fermentation performance, solvent concentrations, acids, biomass concentration, pH and sugar utilization are presented in Fig. 4(a–d). It was noticeable that the steady state occurred when continuous fermentation of SADRB was performed at dilution rates of 0.01, 0.02 and 0.03 h^−1^ and could be stably maintained during 300 h of continuous fermentation. While with a dilution rate of 0.05 h^−1^ the solvent production reached a maximum after 48 h of 8.33 g/L, it started decreasing along with fermentation time thereafter (Fig. 4d). Ni *et al*., found that the continuous fermentation of butanol by *Clostridium saccharobutylicum* enters a steady state when a stable solvent concentration is obtained in the fermentation period between 100–200 h^34^.

Concentrations of ABE, butanol, acetone, and ethanol, as well as yields and productivities of ABE and butanol, are listed in Table 2 for all tested dilution rates using SADRB. Among all the tested dilution rates, a dilution rate of 0.02 h^−1^ produced the highest production rates of ABE and butanol, at 11.18 and 5.89 g/L, respectively.

**Table 2:** Performance of batch and continuous fermentation of butanol production by *C. acetobutylicum* YM1.

Parameters	Batch fermentation		Continuous fermentation					
	SADRB	Detoxified SADRB	Glucose (5%)	Non detoxified SADRB				Detoxified SADRB
Dilution rate (h^−1^)	—	—	0.05	0.01	0.02	0.03	0.05	0.02
ABE (g/L)	11.92	12.62	16.51	6.17	11.18	9.93	8.33	12.42
Butanol (g/L)	7.53	8.27	9.28	3.63	5.89	5.21	4.51	6.87
Acetone (g/L)	3.67	3.65	6.65	2.14	4.37	4.34	3.46	4.63
Ethanol (g/L)	0.72	0.7	0.58	0.42	0.92	0.57	0.36	0.92
ABE productivity (g/L.h)	0.166	0.175	0.823	0.062	0.224	0.298	0.417	0.248
Butanol productivity (g/L.h)	0.105	0.115	0.464	0.036	0.118	0.156	0.226	0.136
B:A ratio	2.05	2.27	1.4	1.7	1.35	1.2	1.3	1.5
ABE yield (g/g)	0.38	0.32	0.48	0.17	0.34	0.28	0.29	0.43
Butanol yield (g/g)	0.25	0.21	0.27	0.10	0.18	0.15	0.16	0.24

In continuous fermentation, it was found that high solvent productivity could be obtained at low dilution rates^35^. Higher dilution rates were reported to be more suitable for bacterial biomass concentrations, while lower dilution rates were found to be optimal for ABE and butanol production in single-stage continuous fermentation^33^. In our study, we found that increasing the dilution rate to more than 0.02 h^−1^ resulted in lower concentrations of solvent and butanol, which was in agreement with that reported by Godin and Engasser^35^.

Continuous fermentation of SADRB at a dilution rate of 0.05 h^−1^ showed poor performance and the steady-state stage was shorter than that observed with other employed dilution rates. The bacterial biomass concentration and butanol production declined along with the fermentation time (Fig. 4d). The instability of the fermentation at this dilution rate can be attributed to a decline in bacterial biomass concentration, which is also likely due to the destruction of bacterial cells during the fermentation. The maximum ABE and butanol concentrations obtained at 48 h were 8.33 g/L and 4.51 g/L, respectively, and the concentrations of ABE and butanol decreased after 48 h. Sugars were consumed efficiently at the first 48 h and after that, an average of 15 ± 1 g/L sugars remained (Fig. 4d). Accordingly, the suitable dilution rate that maximized ABE and butanol production from SADRB hydrolysate in single-stage continuous fermentation was 0.02 h^−1^.

In continuous fermentation of SADRB, it was observed that increasing the dilution rates led to a decrease in the butanol to acetone ratio (B:A), and the highest ratio of B:A was obtained at a dilution rate 0.01 h^−1^ (Table 2). The decrease of B:A at higher dilution rates can be attributed to the fact that at high dilution rates the dominant bacterial cells are in log phase where more acids are produced, and reutilization of these acids is associated with acetone production^36,37^. Similar results were found by Liew *et al*., when they increased the dilution rate of continuous fermentation of sago starch from 0.03 to 0.22 h^−1^, and a decrease in B:A ratios was observed, with the highest B:A obtained being 1.55^33^.

### Continuous fermentation of detoxified-SADRB

Detoxified SADRB was employed for butanol fermentation in the continuous mode with the strain *C. acetobutylicum* YM1 at a dilution rate of 0.02 h^−1^. The detoxified SADRB contained 40.17 g/L total sugars, and the sugars detected were 24.14 g/L glucose (60.87%), 14.58 g/L xylose (35.59%) and 1.45 g/L cellobiose (3.54%).

**Figure 5:**
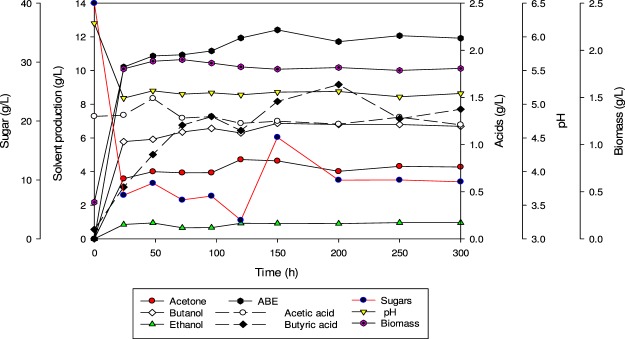
Profiles of single stage continuous fermentation for butanol production from detoxified SADRB hydrolysate using *C. acetobutylicum* YM1 at a dilution rate of 0.02 h^−1^.

A continuous fermentation time-course of detoxified SADRB is shown in Fig. 5. It can be seen that the butanol concentration reached a high concentration after 24 h and continued at a steady state until the fermentation was stopped after 300 h. The bacterial biomass concentration was constant in stationary phase and no decrease in biomass concentration was observed during the fermentation. In addition, the bacterial biomass concentration in the detoxified SADRB culture was higher compared to that found when non-detoxified SADRB was employed (Figs 4b and 5). The results demonstrated that detoxification of SADRB by charcoal reduced fermentation inhibitors and then allowed the cells to grow better, which indicated that detoxification is essential for enhanced ABE fermentation performance. Continuous fermentation of detoxified SADRB produced a total of 12.42 g/L ABE containing 6.87 g/L butanol, 4.63 g/L acetone and 0.92 g/L ethanol and giving an ABE yield and productivity of 0.43 g/g and 0.428 g/L.h, respectively.

Compared to non-detoxified SADRB, in which lower ABE and butanol were produced, detoxified SADRB resulted in higher concentrations of ABE and butanol, which most likely was due to the removal of inhibitory compounds that cause cell inhibition and subsequently lower ABE fermentation efficiency. Detoxification by activated charcoal has been used previously for the removal of fermentation toxic compounds from different biomass hydrolysates^38–41^. Activated charcoal showed that it has good efficiency for the removal of fermentation inhibitors and it can be regenerated after detoxification^40^.

Table 3 compares continuous butanol production from SADRB and glucose in this study and the results reported in the literature. The results of this study showed that *C. acetobutylicum* YM1 has high efficiency for butanol production in continuous fermentation from both substrates, glucose and SADRB hydrolysate.

**Table 3:** Comparison of continuous fermentation of butanol from different substrates.

Substrate	Strain	Dilution rate (h^−1^)	ABE (g/L)	Butanol (g/L)	ABE productivity (g/L.h)	Reference
Corn stover	*C. saccharobutylicum* DSM 13864	0.15	11.43	7.81	0.429	^34^
Cane molasses	*C. saccharobutylicum* DSM 13864	0.1	11.74	7.18	0.294	^34^
Sago starch	*C. saccharobutylicum* DSM 13864	0.05	9.10	5.19	0.455	^33^
Corn starch	*C. beijerinckii* BA101	0.02	7.20	—	0.144	^43^
De-fibrated-sweet potato-slurry	*C. acetobutylicum* P-262	0.129	7.73	5.52	1.00	^53^
Glucose (6%)	Immobilized *C. acetobutylicum*	0.05	11.74	7.80	0.630	^42^
Glucose (5%)	*C. acetobutylicum* YM1	0.05	16.51	9.28	0.823	This study
Deoiled rice bran	*C. acetobutylicum* YM1	0.02	12.42	6.87	0.248	This study

Based on the data presented in Table 3, ABE and butanol produced by *C. acetobutylicum* YM1 in continuous fermentation using 5% glucose was satisfactorily higher than ABE and butanol as reported by Dolejs *et al*. in the continuous fermentation of immobilized *C. acetobutylicum* fed with a glucose concentration of 60 g/L under the same dilution rate of 0.05 h^−1^ ^42^. Additionally, a higher ABE productivity of 0.823 g/L.h was obtained in this study compared to the 0.63 g/L.h ABE productivity found by Dolejs *et al*.^42^.

Moreover, continuous fermentation of detoxified SADRB by *C. acetobutylicum* YM1 at a dilution rate of 0.02 h^−1^ resulted in the production of 12.42 ABE and 6.87 g/L butanol with a productivity of 0.248 g/L.h, which was higher than that found by Ezeji *et al*.^43^ when corn starch was used as a substrate in continuous fermentation by *C. beijerinckii* BA101 (Table 3). Continuous fermentation of dilute acid pretreated-corn stover at a dilution rate of 0.15 h^−1^ conducted by Ni *et al*.^34^ showed lower concentration of ABE but higher ABE productivity compared to that obtained in our study using SADRB hydrolysate.

According to the results that were summarized in Table 2, it can be seen that the productivities and yields of ABE and butanol in continuous fermentation were higher than that obtained under the batch fermentation process. The ABE and butanol productivities gained in continuous fermentation using glucose as a substrate were approximately 5 times higher than that obtained from batch fermentation of SADRB. In a similar trend, the ABE and butanol productivities found when detoxified SADRB was consumed in continuous fermentation were 2.6 times and 1.3 times higher, respectively, compared to that found using batch fermentation.

The continuous fermentation process is preferable in industry due to the high productivity and lower preparation time^44^. For the further improvement of butanol fermentation performance, applying *in situ* continuous fermentation with a product recovery approach has been suggested^10,45,46^. Applying process integration systems for butanol production is expected to reduce the cost of capital and operations and therefore, makes butanol production an economically efficient process^10,47,48^. An integrated system of simultaneous saccharification, fermentation and product recovery for butanol production from corn stover by *Clostridium beijerinckii* P260 was reported to be an efficient high butanol productivity of 0.19 g/L.h, while the butanol productivity without product recovery was 0.12 g/L.h^47^. An efficient process integration for butanol production from whey permeate and recovery by gas stripping using immobilized cells of *C. acetobutylicum* P262 was reported by Qureshi and Maddox^49^. This integrated system was operated in continuous fermentation for 4 months in steady rate operation and high productivity of butanol. *In situ* two-stage gas stripping recovery process was integrated in ABE fermentation for butanol production by *Clostridium acetobutylicum* JB200 using a fibrous bed bioreactor was found to be highly productive for butanol production compared to fermentation without *in situ* gas stripping^10^. The reported integrated process was also effective in production of high butanol concentration and thus the process was energy saving and more economic^48^.

This study was carried out in a single stage continuous fermentation with free cells of *C. acetobutylicum* YM1, but for higher butanol productivities, the use of a two-stage continuous fermentation or continuous fermentation using immobilized cultures or biomass-retention cultures are the best choices that have been proposed for higher butanol productivities due to the possibility of sustaining higher dilution rates^50,51^.

## Economic Study

**Figure 6:**
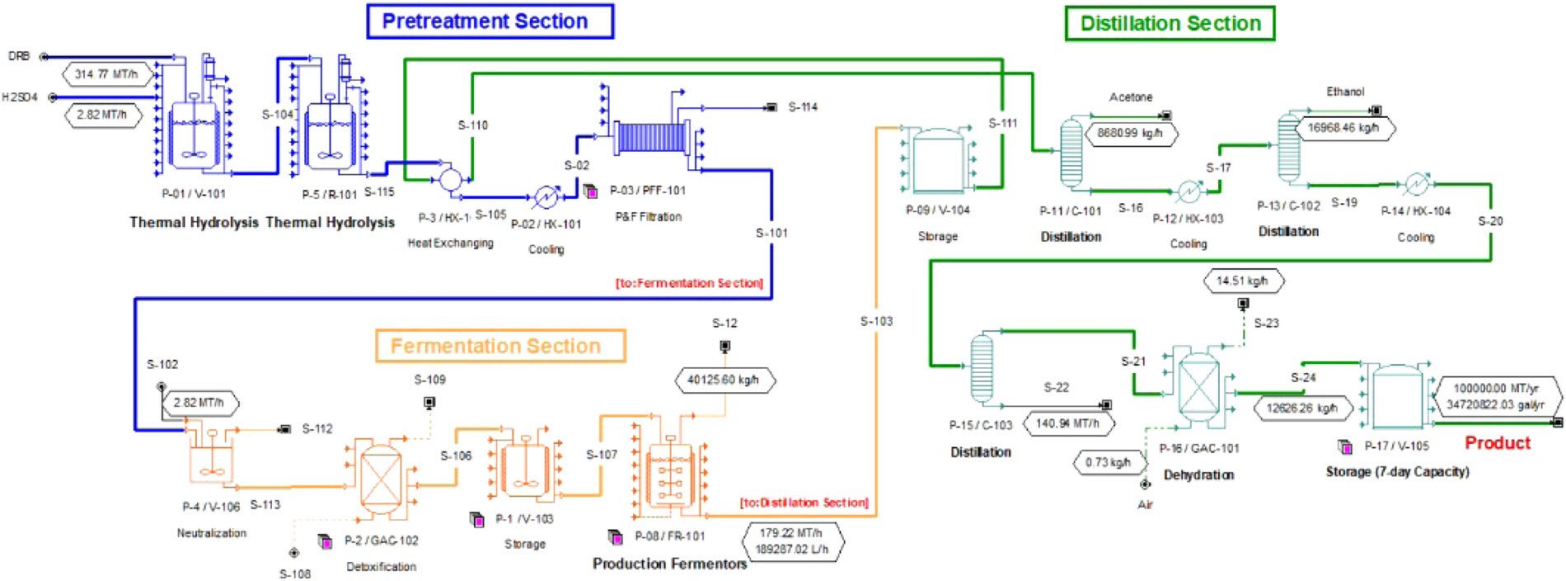
Process flow diagram for continuous butanol fermentation of DRB.

Figure 6 shows the process flow diagram of the plant generated using SuperPro Designer. The process starts with pretreating the DRB with dilute sulfuric acid in two thermal reactors in series. The pretreated DRB will be filtered by passing through a filter press before neutralization. Detoxification column filled with activated carbon will be installed prior to fermentation to remove any inhibitors formed during dilute acid pretreatment. The product will proceed to fermentation process. The reaction will be conducted in a fermenter with seed feeding of *C. acetobutylicum* YM1. Four major components will be obtained from the fermenter, *i.e*. butanol, acetone, ethanol and water. Butanol is the desired product with acetone and ethanol as by-products. They will be separated using three distillation columns in view of their different boiling points. In the first distillation column, acetone will be collected as top product, the bottom product will pass through second distillation column and ethanol will be separated as top product. Finally, butanol and water will pass through the third distillation column and butanol will be collected as top product. Dehydration of butanol will be performed to concentrate the product.

Economy analysis was performed for this plant setup and considering the annual butanol production capacity of 100 × 10^6^ kg from DRB with 330 operating days (equivalent to 7920 h/year). Table 4 shows the fixed capital estimate for production of butanol from DRB. For this plant, total equipment cost was estimated to be $32,385,000. Other significant costs such as installation, process piping, instrumentation, insulation, electrical, buildings, yard improvement and auxiliary facilities were listed in Table 4. The total plant direct cost (TPDC) was projected to be $105,652,000. The total plant indirect cost (TPIC) which includes engineering and construction was $63,391,000 in total, whereby engineering and construction contributed $26,413,000 and $36,978,000, respectively. The total plant cost (TPC) which is the summation of TPDC and TPIC was $169,043,000. Contractor’s fee and contingency (CFC) was $25,357,000 (Contractor’s fee = $8,452,000; Contingency = $16,904,000). These make up the direct fixed capital cost (DFC) of $194,400,000.

**Table 4:** Fixed capital estimate for production of butanol from de-oiled rice bran.

**A. Total plant direct cost (TPDC; physical cost)**	**($)**
Equipment purchase cost	32,385,000
Installation	12,383,000
Process piping	11,335,000
Instrumentation	12,945,000
Insulation	972,000
Electrical	3,239,000
Buildings	14,573,000
Yard improvement	4,858,000
Auxiliary facilities	12,954,000
**TPDC**	**105,652,000**
**B. Total plant indirect cost (TPIC)**	
Engineering	26,413,000
Construction	36,978,000
**TPIC**	**63,391,000**
**C. Total plant cost (TPC = TPDC + TPIC)**	**169,043,000**
**D. Contractor’s fee and contingency (CFC)**	
Contractor’s fee	8,452,000
Contingency	16,904,000
**CFC**	**25,357,000**
**E. Direct fixed capital cost (DFC = TPC + CFC)**	**194,400,000**

Table 5 shows the annual operating cost including raw materials, facility and utilities costs. Two different prices of DRB were chosen to estimate the annual operating cost, *i.e*. DRB at unit price of $50/MT (case 1) and $20/MT (case 2). Approximately 1,246,498 MT of DBR will be used annually. With the basis of DRB unit cost $50 and $20 per MT, annual cost is estimated to be $62,324,922 (for case 1) and $24,929,969 (for case 2), respectively. Apart from DRB, sulfuric acid and sodium hydroxide are the materials used in the plant. This contributed to a total raw material cost of $65,849,436 (case 1) and $28,454,483 (case 2) per year. Cost of facilities and utilities also considered in annual operating cost, as presented in Table 5. The plant projected to consume 27,727,819 kW-h electricity in a year. A unit cost of electivity is $0.01 per kW-h, making an annual electricity cost of $2,772,782. Other utilities are steam, cooling water and chilled water with usage of 1,813,483 MT, 179,715,372 MT and 8,465,034 MT, respectively. With unit price of $12.00, $0.05 and $0.01 per MT, total cost of steam, cooling water and chilled water were $21,761,793, $8,985,769 and $3,386,014, respectively. This make up a total utilities cost of $36,906,357.

**Table 5:** Annual operating cost for butanol production from de-oiled rice bran.

	Unit cost ($)	Annual amount	Cost ($/year)
**A. Raw materials ($/MT)**			
De-oiled rice bran (case 1)	50.000	1,246,498	62,324,922
De-oiled rice bran (case 2)	20.000	1,246,498	24,929,969
Sulfuric acid	70.000	22,347.34	1,564,314
Sodium hydroxide	100.000	19,602	1,960,200
**Total (case 1)**			**65,849,436**
**Total (case 2)**			**28,454,483**
B. Facility ($/kg MP)*	**366.108**		**36,610,776**
**C. Utilities**			
Standard power ($/kW-h)	0.010	27,727,81	2,772,782
Steam ($/MT)	12.00	1,813,483	21,761,793
Cooling water ($/MT)	0.05	179,715,372	8,985,769
Chilled water ($/MT)	0.01	8,465,034	3,386,014
**Total**			**36,906,357**

*MP - Total flow of main product (butanol).

Having both fixed capital cost and annual operating cost estimated, the total investment cost to operate this plant is projected to be $213,567,000 for case 1 and $210,168,000 for case 2. Table 6 shows the profitability analysis of the plant. The annual production of butanol, acetone and ethanol produced is projected to be 100,000,000 kg, 68,753,445 kg and 134,390,206 kg. With the basis of $1.48 (butanol), $0.959 (acetone) and $0.90 (ethanol) unit price, it is estimated to generate annual revenue of $334,885,739. With these analyses, the unit production cost of butanol is $1.405/kg for case 1 and $1.031/kg for case 2.

**Table 6:** The profitability analysis of production of butanol from de-oiled rice bran in continuous fermentation process.

Revenue	Amount produced (kg/yr)	Unit selling cost ($/kg)	Annual revenue ($/yr)
Butanol	100,000,000	1.48	148,000,000
Acetone	68,753,445	0.959	65,934,554
Ethanol	134,390,226	0.90	120,951,186

## Conclusion

Continuous fermentation for butanol production SADRB was successfully performed. Further detoxification of SADRB by activated charcoal significantly reduced fermentation inhibitory compounds and improved the continuous fermentation performance. Pretreatment of de-oiled rice bran followed by detoxification was a necessary process for the enhancement of butanol production. The highest ABE (12.42 g/L), butanol (6.87 g/L) and ABE productivity (0.428 g/L.h) were obtained when detoxified SADRB was utilized in continuous fermentation at a dilution rate of 0.02 h^−1^ using *C. acetobutylicum* YM1. The results indicate that continuous fermentation using DRB hydrolysate is a potential as an inexpensive substrate for the high productivity of butanol production. This study presented economic analysis of continuous conversion process of DRB to butanol by *C. acetobutylicum* YM1. The process is including dilute acid pretreatment of DRB, detoxification of sugars released from the pretreatment, fermentation and recovery of butanol, acetone and ethanol by distillation process. Based on the data from this study, the cost of butanol production estimated as $1.405/kg based on DRB price of $50/MT while in case the DRB price drop to $20/MT, this would reduce the cost of butanol production to $1.031/kg.

## Methods

### Microorganism

In this study, a local aerotolerant strain of *Clostridium acetobutylicum* YM1 was used. The inoculum was prepared by activating a spore suspension (1 mL) in 10 mL of a tryptone-yeast extract-acetate medium (TYA) with a subsequent heat shock for 1 min in boiling water, cooling in ice water and then incubation for 1–2 days at 30 °C under anaerobic condition. Before inoculation, the TYA medium was sparged with nitrogen gas (95%) to facilitate anaerobic conditions.

The inoculum was prepared using TYA medium that consisted of 20 g/L glucose, 6 g/L tryptone, 3 g/L ammonium acetate, 2 g/L yeast extract, 0.5 g/L KH_2_PO_4_, 0.3 g/L MgSO_4_.7H_2_O, and 0.01 g/L FeSO_4_.7H_2_O.

### Pretreatment of de-oiled rice bran (DRB)

Rice bran was obtained from the Abidin Rice Mill Sdn. Bhd., Perlis, Malaysia, and kept at 4 °C until use. Rice bran was de-oiled by extracting the oil from rice bran using hexane (J.T. Baker Chemical Co. Phillipsburg, NJ, USA), as reported by Al-Shorgani *et al*.^13^. The pretreatment with sulfuric acid was carried out by soaking 12% (w/v) of DRB in a 1% (v/v) sulfuric acid solution and then autoclaving it (at 121 °C/15 psi) for 1 h. The solid materials after pretreatment were separated by filtration and the pH of the pretreated DRB with sulfuric acid (SADRB) was adjusted to 6.2 by using 10 M NaOH.

### Detoxification of SADRB hydrolysate

Detoxification of the SADRB hydrolysate was applied in order to reduce the concentration of inhibitory compounds such as furfural, HMF, acetic acid, formic acid, and levulinic acid. The SADRB hydrolysate (pH 6) was passed through activated charcoal that was packed in a glass column (60 cm × 2 cm). Ten grams of activated charcoal was used to detoxify 1 L of SADRB hydrolysate. The pH of the detoxified SADRB was adjusted again to a pH of 6.2 before sterilization.

### Fermentation

**Figure 7:**
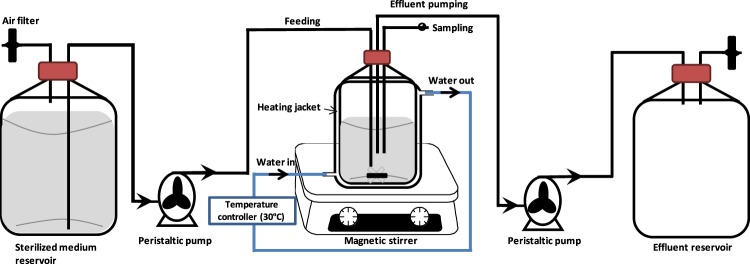
Schematic diagram of the continuous butanol fermentation.

Batch fermentation experiments were conducted in 100-mL serum bottles outfitted with rubber stoppers and crimped with aluminium seals, with a working volume of 80 mL under anaerobic condition. Continuous fermentation was conducted in a 1 L bioreactor (jacketed-Scott Duran bottle) with a working volume of 600 mL. The jacketed-bioreactor vessel was heated by cycling water continuously in the jacket at 30 °C. The medium was pumped at various dilution rates using a peristaltic pump (Masterflex, HV-77120-42, Cole-Parmer Instrument Co., Vernon Hills, IL, USA). Figure 7 shows the schematic diagram of the bioreactor and continuous fermentation system.

Sterilized SADRB was used as a fermentation medium and stored in a feeding tank that was connected to the fermentation bioreactor. The bioreactor was inoculated using a 10% (v/v) fresh inoculum of *C. acetobutylicum* YM1 (grown for 20 h) and incubated at 30 °C. The bioreactor was heated at a constant temperature (30 °C) during the continuous fermentation by cycling water in the jacket of the bioreactor. Continuous fermentation was started by feeding SADRB medium at certain flow rates after 24 h of fermentation, in which the bacteria reached stationary phase and entered the solventogenic phase. The volume of the fermentation medium in the bioreactor was kept constant by using the peristaltic pump.

Continuous fermentation was initiated after 24 h of batch fermentation to allow significant bacterial biomass concentration and butanol production. After that, fresh SADRB medium was fed into the fermentor, and the volume of the fermentation medium in the fermentor was maintained at a constant by setting a purge flow with the same volumetric stream rate as the feed flow rate. No nitrogen gas was fed into the culture during the continuous fermentation, and the pH was not controlled during the continuous fermentation. Samples were collected periodically for fermentation monitoring and analysis.

The SADRB hydrolysate was supplemented with TYA ingredients (without glucose) and the pH of the medium was adjusted to 6.2 before sterilization. The fermentation medium was mixed and stirred during the continuous fermentation using a magnetic stirrer at 150 rpm.

### Analysis methods

Fermentation samples were collected and centrifuged at 5000 g for 5 min, and the supernatant was used for the analysis of solvents, acids and sugars.

The analysis of acetone, butanol, ethanol, acetic acid and butyric acid was performed using a gas chromatograph (7890A GC-System, Agilent Technologies, Palo Alto, CA, USA) equipped with a flame ionization detector (FID) and a 30-m capillary column (Equity-1; 30 m × 0.32 mm × 1.0 µm film thickness; Supelco Co., Bellefonate, PA, USA). The injection temperature was set at 250 °C and the detection temperature was set at 280 °C. The carrier gas used was helium at a flow rate of 1.5 mL/min.

Inhibitory compounds such as furfural, HMF, acetic acid, formic acid, and levulinic acid were measured using a high-performance liquid chromatograph (HPLC; 12000 Series, Agilent Technologies, Palo Alto, CA, USA). Separations and concentrations were performed on a Phenomenex C18 column (250 × 4.6 mm ID; Phenomenex Inc., Torrance, CA, USA) using a UV detector at 220 nm (UV-D; 1200, Agilent Technologies, Palo Alto, CA, USA) at 40 °C. The mobile phase was a mixture of 95% sulfuric acid (20 mM) and 5% acetonitrile, with an overall flow rate of 1 mL/min.

Sugars including glucose, xylose and cellobiose were estimated by HPLC (12000 Series, Agilent Technologies, Palo Alto, CA, USA) using a Shodex Asahipak NH2P-50 4E column (4.6 mm ID × 250 mm; Shodex, Kanagawa, Japan). Sugar concentrations were measured with a refractive index detector (RID; 1200, Agilent Technologies, Palo Alto, CA, USA) at 30 °C with a mobile phase flow rate of 1 mL/min, with a mixture of acetonitrile (60%) and water (40%).

The concentrations of total reducing sugars were measured using the 3,5-dinitrosalicylic acid (DNS) assay according to Miller’s method^52^. The bacterial biomass concentration was estimated as dry cell weight (DCW).

The volumetric productivity for ABE or butanol in batch fermentation was calculated according to the Equation 1 while the volumetric productivity of ABE or butanol in continuous fermentation was calculated by Equation 2. Equation 3 was used to define the yield of ABE and butanol.

1 $${\rm{Productivity}}\,{\rm{of}}\,{\rm{ABE}}\,{\rm{or}}\,{\rm{butanol}}\,({\rm{g}}/{\rm{L}}.{\rm{h}})={\rm{Concentration}}\,{\rm{of}}\,{\rm{ABE}}\,{\rm{or}}\,{\rm{butanol}}\,({\rm{g}}/{\rm{L}})/{\rm{Fermentation}}\,{\rm{time}}\,({\rm{h}})$$

2 $${\rm{Productivity}}\,{\rm{of}}\,{\rm{ABE}}\,{\rm{or}}\,{\rm{butanol}}\,({\rm{g}}/{\rm{L}}.{\rm{h}})={\rm{Concentration}}\,{\rm{of}}\,{\rm{ABE}}\,{\rm{or}}\,{\rm{butanol}}\,({\rm{g}}/{\rm{L}})\times {\rm{Dilution}}\,{\rm{rate}}\,({h}^{-1})$$

3 $${\rm{Yield}}\,{\rm{of}}\,{\rm{ABE}}\,{\rm{or}}\,{\rm{butanol}}\,({\rm{g}}/{\rm{g}})={\rm{Concentration}}\,{\rm{of}}\,{\rm{ABE}}\,{\rm{or}}\,{\rm{butanol}}\,({\rm{g}}/{\rm{L}})/{\rm{Concentration}}\,{\rm{of}}\,{\rm{sugar}}\,{\rm{consumed}}\,({\rm{g}}/{\rm{L}})$$

## Financial Evaluation

The production of butanol was simulated in SuperPro Designer (version 8.5003, Intelligen Inc.) for basis of 330 days/year. In the simulation, material and energy balance was computed with the basis of annual butanol production of 100,000 MT. The economic assessment was conducted in SuperPro economic evaluation where the butanol is produced continuously in a fermenter as main product and acetone and ethanol are by-products. The price value and calculation were based on year 2018. In this analysis, site development, transportation and mechanical pretreatment of DRB were not included.

## Supplementary information

Author List Changes Approval form_SREP-18-02725C
