# Supplementary material for: Continuous Butanol Fermentation of Dilute Acid-Pretreated De-oiled Rice Bran by Clostridium acetobutylicum YM1
Source: Sci Rep. 2019 Mar 15;9:4622. doi: 10.1038/s41598-019-40840-y (PMC6420626; doi:10.1038/s41598-019-40840-y)
Supplement: Supplementary file 1 — Author List Changes Approval form_SREP-18-02725C [file 41598_2019_40840_MOESM1_ESM.pdf]

nature publishing group npg

In accordance to Nature Publishing Groups Authorship Policy we agree to change the authors of the manuscript as indicated below.

NAME OF JOURNAL: Scientific Reports

TITLE OF MANUSCRIPT: Continuous Butanol Fermentation of Dilute Acid-Pretreated De-oiled Rice Bran by Clostridium acetobutylicum YM1

MANUSCRIPT NUMBER: SREP-18-02725C

CORRESPONDING AUTHORS NAME: Mohd Sahaid Kalil & Najeeb Kaid Nasser Al-Shorgani

PREVIOUS AUTHOR NAMES:
Najeeb Kaid Nasser Al-Shorgani, Abdualati Ibrahim Al-Tabib, Abudukeremu Kadier, & Mohd Sahaid Kalil

UPDATED AUTHOR NAMES:
Najeeb Kaid Nasser Al-Shorgani, Abdualati Ibrahim Al-Tabib, Abudukeremu Kadier, Mohd Fauzi Zanil, Kiat Moon Lee & Mohd Sahaid Kalil

CHANGE TO AUTHOR LIST: In the second round revision, the reviewers asked for economic analysis to improve the manuscript. The economic analysis has been performed by Mohd Fauzi Zanil and Kiat Moon Lee. Therefore, these two authors have been added as they contributed to this manuscript.

Print Name	Signature	Date
Najeeb Kaid Nasser Al-Shorgani	[signature]	11/11/2018
Abdualati Ibrahim Al-Tabib	[signature]	11/11/2018
Abudukeremu Kadier	[signature]	12/11/2018
Mohd Fauzi Zanil	[signature]	11/11/2018
Kiat Moon Lee	Kiatmoon	11th Nov 2018
Mohd Sahaid Kalil	[signature]	12/11/2018
